# Overcoming Nitrogen Reduction to Ammonia Detection Challenges: The Case for Leapfrogging to Gas Diffusion Electrode Platforms

**DOI:** 10.1021/acscatal.2c00888

**Published:** 2022-04-28

**Authors:** Martin Kolen, Davide Ripepi, Wilson A. Smith, Thomas Burdyny, Fokko M. Mulder

**Affiliations:** † Materials for Energy Conversion and Storage (MECS), Department of Chemical Engineering, Faculty of Applied Sciences, Delft University of Technology, van der Maasweg 9, 2629 HZ Delft, The Netherlands

**Keywords:** ammonia, detection, nitrogen reduction, gas diffusion electrode, catalyst

## Abstract

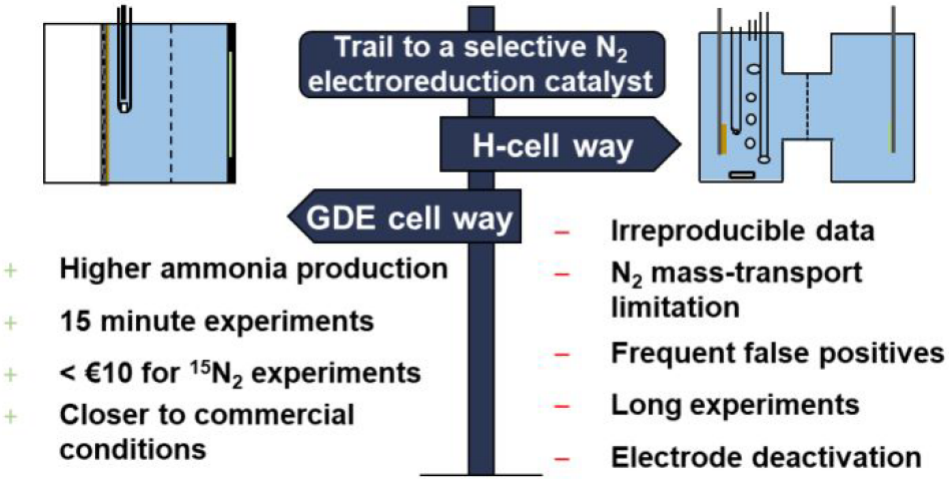

The nitrogen reduction reaction (NRR) is a promising pathway toward the decarbonization of ammonia (NH_3_) production. However, unless practical challenges related to the detection of NH_3_ are removed, confidence in published data and experimental throughput will remain low for experiments in aqueous electrolyte. In this perspective, we analyze these challenges from a system and instrumentation perspective. Through our analysis we show that detection challenges can be strongly reduced by switching from an H-cell to a gas diffusion electrode (GDE) cell design as a catalyst testing platform. Specifically, a GDE cell design is anticipated to allow for a reduction in the cost of crucial ^15^N_2_ control experiments from €100–2000 to less than €10. A major driver is the possibility to reduce the ^15^N_2_ flow rate to less than 1 mL/min, which is prohibited by an inevitable drop in mass-transport at low flow rates in H-cells. Higher active surface areas and improved mass transport can further circumvent losses of NRR selectivity to competing reactions. Additionally, obstacles often encountered when trying to transfer activity and selectivity data recorded at low current density in H-cells to commercial device level can be avoided by testing catalysts under conditions close to those in commercial devices from the start.

## Introduction

Novel electrochemical reactions provide hope for a scalable means of storing intermittent electricity within chemical bonds, simultaneously aiding in the buffering of renewable energy while providing a route for offsetting carbon-based fuels. Nitrogen reduction electrochemistry in particular has the potential to directly offset 1–1.4% of global CO_2_ emissions currently emitted during ammonia (NH_3_) production, with additional potential for using ammonia as an energy carrier in further applications (e.g., shipping, aviation).^1−3^ Such promise has led to a large number of researchers entering the nitrogen electrochemistry field in recent years, with substantial effort placed on developing selective catalysts capable of driving nitrogen reduction to ammonia over the more favorable hydrogen evolution reaction (HER).^4^ If parallel electrochemical reactions with low solubility gaseous reagents are taken as precedent (e.g., electrochemical reduction of CO_2_ and O_2_), once high selectivity catalysts have been identified, there are established approaches for increasing reaction rates, reducing overpotential, increasing stability, and eventually incorporating promising catalysts supported on a high surface area support such as a gas-diffusion electrode (GDE) in commercial devices.^5,6^ However, currently the academic field appears to be at a standstill because, due to the inefficiency of the reaction in aqueous electrolyte, no selective N_2_ reduction catalyst has been conclusively presented, yet.^7,8^

The difficulty of achieving dominant faradaic efficiencies (FE) for the nitrogen reduction reaction (NRR) is commonly attributed to the slow kinetics of breaking the nitrogen triple bond in a 6-electron transfer process compared to only 2-electron transfers for HER. Any catalyst then needs to balance the simultaneous challenge of improving the kinetics for N_2_ reduction while suppressing HER.^4^ A large body of knowledge is available on altering the selectivity of electrocatalytic reactions including strategies like alloying, doping, or introducing defects.^9^ For example, the selectivity of the CO_2_ reduction reaction (CO_2_RR) can be tuned toward ethanol (from 30 to 41% FE) or ethylene (from 66 to 80% FE) by alloying Cu with Ag or Al, respectively.^10,11^ Extensive exploration of such strategies might yield a selective catalyst for NRR, too.

Despite the wealth of electrochemical expertise entering this novel research field, substantial detection challenges have persisted. Typical experiments in aqueous electrolyte produce μM levels of NH_3_ and NH_4_^+^, which are on par with common NH_3_ contamination levels.^7,8,12^ Adventitious NH_3_/NO_*x*_ often found in membranes, catalyst precursors, electrolytes, and N_2_ feedstocks commonly occur at concentrations between 2 and 20 μM in the electrolyte (see Table S2).^13−17^ To highlight this issue, we calculate the accumulated ammonia in the electrolyte for an NRR partial current of 100 μA, which is among the highest reported rates in literature. Even at this high rate, the accumulated ammonia in the electrolyte after 30 min of electrolysis without catalyst deactivation (electrolyte volume: 30 mL) is only 20.7 μM, which is too close to common contamination levels for unambiguous quantification.

These measurement challenges have led to false positive measurements of NRR activity and in some cases to retractions and refutations of publications that were initially believed to be groundbreaking.^7,8,13−19^ To overcome the measurement challenges associated with NRR, extensive experimental protocols were introduced which, if executed correctly, are able to avoid false positives. In accordance with these protocols, it is particularly important to show that the NH_3_ yield from experiments with ^14^N_2_ quantitatively agrees with ^15^N_2_ experiments and that the ^15^N_2_ used in those experiments is free of ^14/15^NH_3_ and ^14/15^NO_*x*_ contamination.^7,12,20^ Critical reviews agree that very few published reports meet these criteria.^7,8,12^ As such, only a small reliable data set is available for potentially invaluable computational studies.^11^ In short, if the challenges of detecting electrochemically produced NH_3_ were removed, there would be an undeniable propulsion forward of the research field due to increased reproducibility and a larger reliable data set.

When analyzing the typical electrochemical NH_3_ synthesis system, however, it becomes clear that researchers face the dilemma of choosing either reliable data or affordable, fast experiments. As we will discuss in more detail below, carrying out reliable protocols is time-consuming and expensive due to the long electrolysis steps involved and the use of expensive ^15^N_2_. We found, that this dilemma is linked to the H-cell-type cell design used in most studies. On the other hand, if the same experiments were performed in a gas diffusion electrode cell, then testing restrictions due to detection challenges have the potential to be overcome. We will show that the compact design of gas diffusion electrode cells and their high N_2_ mass transport, which is decoupled from the gaseous flow rate, makes them advantageous to use for NRR studies.

In this perspective, we provide a technological motivation for leapfrogging catalyst development within H-cells and promoting gas-diffusion electrodes as a substrate for the development of NRR catalysts. We first analyze what limits the progress of the field with H-cells. Then the benefits for NH_3_/NH_4_^+^ detection of supplying N_2_ from a near N_2_ gas-phase are discussed from a system and instrumentation perspective and contrasted to the current approach of supplying N_2_ from the bulk electrolyte. We then argue what other implications the use of higher surface area electrodes and a reduced liquid diffusion pathway have for NRR catalyst screenings. Lastly, we examine the potential and limitations of gas diffusion electrodes as a platform to benchmark NRR catalysts.

### Limits of Product Detection by Configuration and Operating Conditions

**Figure 1:**
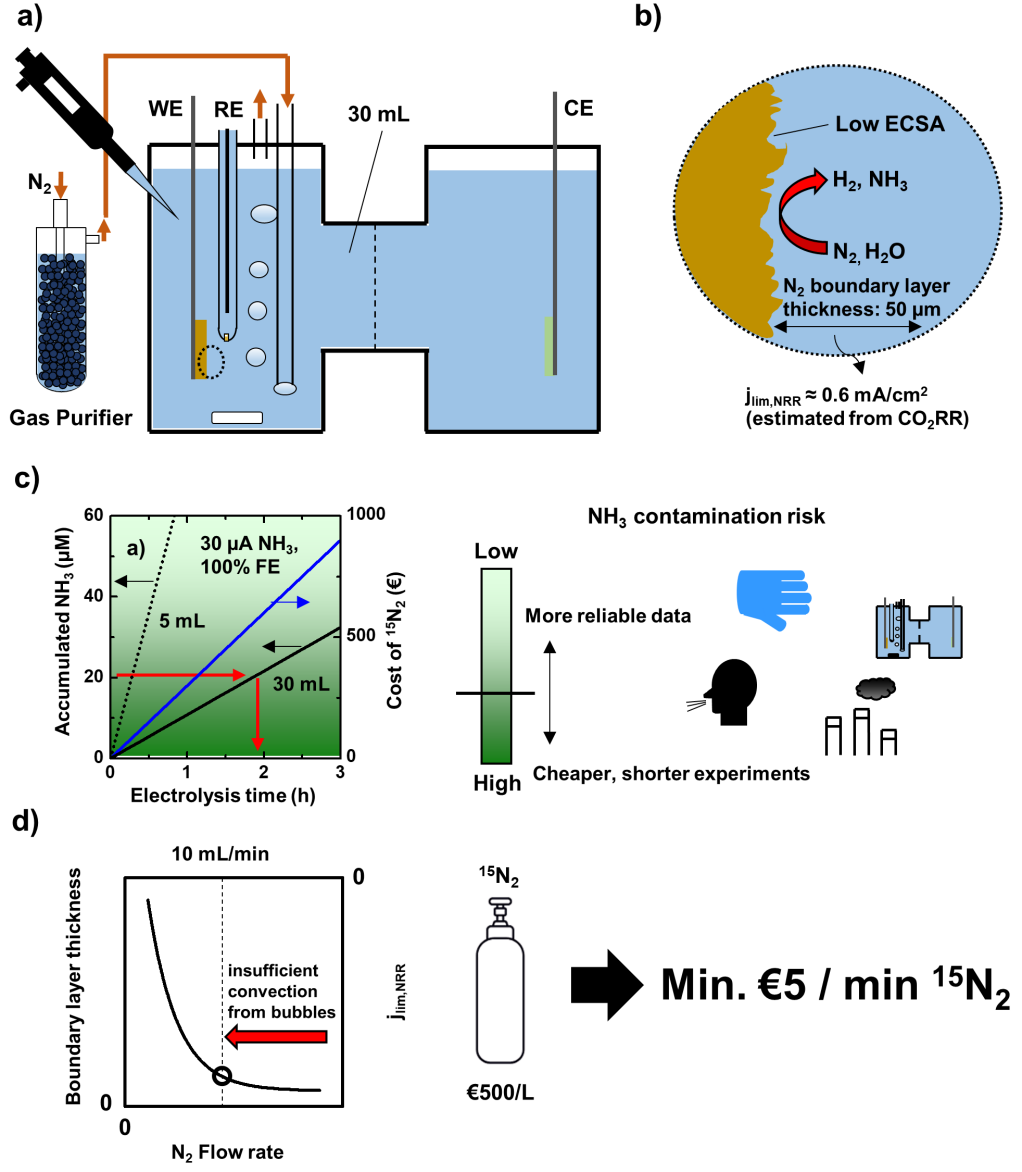
Limitations of H-cell cell design for NRR studies. (a) Schematic of an H-cell and gas purification. (b) Schematic of the electrode surface with 50 μm boundary layer and resulting mass-transport limiting current for NRR j_lim,NRR_. (c) Dependence of the accumulated NH_3_ and the cost of an isotope labeling experiment on the electrolysis time and the electrolyte volume. Ammonia production from NRR: 30 μA, flow rate during ^15^N_2_ experiments: 10 mL/min. The green color gradient represents the risk of NH_3_ contamination (summarized from Table S2). (d) Dependence of N_2_ mass transport to the cathode on the N_2_ flow rate and resulting minimal cost of isotope labeling. Adapted from ref (26). Copyright 2018 American Chemical Society.

The most commonly used electrochemical cells for NRR are H-cells which are comprised of a working, reference, and counter electrode submerged in two electrolyte-filled compartments separated by a membrane (see Figure 1a). The N_2_ is supplied by bubbling into the electrolyte while stirring. NH_3_ production is typically quantified from liquid samples of the electrolyte.

Using H-cells for NRR studies leads to several limitations which are illustrated in Figure 1b–d. Electrodes in H-cells have low electrochemical surface area (ECSA) for NRR (Figure 1b) which makes them prone to deactivation for example due to deposition of impurities from the electrolyte on the electrode surface.^21,22^ In addition, the only marginally water-soluble nitrogen gas has to be supplied from the bulk electrolyte which leads to a relatively large boundary layer thickness and therefore low mass transport.^23^ These two limitations will be discussed in greater detail later in this perspective.

In Figure 1c, we compare the NH_3_ production from NRR with commonly observed NH_3_/NO_*x*_ contamination levels. To quantify NH_3_/NO_*x*_ contamination levels, we summarized the available literature on the magnitude of different contamination sources in Table S2. Most contamination sources are in the range of 2–20 μM, but up to 150 μM of NH_3_ is possible. Reports of NH_3_ contamination with very sensitive and selective detection methods have shown that an NH_3_ background of 0.5–2 μM cannot be removed, even with extensive cleaning.^7,16,24^ Therefore, we propose that the risk of NH_3_ contamination is highest if the NH_3_ production from NRR does not exceed 2 μM and gradually decreases with increasing NH_3_ production. This is illustrated using a color gradient in Figure 1c. NH_3_ production from NRR should at least exceed 20 μM to avoid the region with the most contamination sources (2–20 μM). The reported NH_3_ production rates in literature vary from 3 to 300 μA/cm^2^ NRR partial current density.^25^ In the comparison in Figure 1c, we chose a production rate of 30 μA NRR partial current density which we refer to as an intermediate NH_3_ production rate throughout this perspective (unless otherwise noted we will assume an electrode area of 1 cm^2^ in all calculations). With this intermediate production rate and the median electrolyte volume of 30 mL used in H-cell studies (see Table S3), the electrolysis time required to reach 20 μM NH_3_ is 1.6 h (see Figure 1c). Therefore, to reach an NH_3_ concentration that is large enough to at least exceed the most common NH_3_ contaminations, NH_3_ from electrolysis must be accumulated for almost 2 h. Our calculation agrees well with the electrolysis time that is used in practice in NRR studies (median from Table S3: 2h). While such long experiments are necessary for durability tests once an active catalyst has been identified, initial experiments to measure the NRR activity of promising materials should be much shorter to enable fast advancement in NRR research. In addition, shorter experiments reduce the risk of contamination entering the cell, for instance from a not properly purified N_2_ feed gas.

While most NH_3_ contaminations in Table S2 are below 20 μM, this threshold is somewhat arbitrary and accurate quantification is also possible below 20 μM, which comes at the cost of a higher risk of false positives that must be reduced with more frequent control experiments and more extensive cleaning steps the lower the NH_3_ concentration gets. Due to the unavoidable NH_3_ background of 0.5–2 μM in the electrolyte, reports of catalysts that do not exceed this threshold are highly questionable.^7,15,16^ Besides NH_3_ contamination, another factor that can limit the experimental throughput is the detection limit of some NH_3_ analytical methods.

The detection limit of the most commonly used NH_3_ detection method in literature, the indophenol method, is sufficiently low, but the method requires time-consuming sample preparation with unstable reagents, which leads to long bench time. Therefore, the indophenol method is undesirable for NRR research from a practical perspective.^24,27−29^ It is widely accepted that control experiments with ^15^N_2_ which quantitatively agree with ^14^N_2_ experiments are essential to prove that NH_3_ production originated from NRR and not from contamination of either ^14^N and/or ^15^N species.^7,16,24^ The detection of the isotopologue ^15^NH_3_ requires an isotopically selective detection method, which in most cases is liquid state ^1^H NMR that can detect the triplet and doublet ^1^H spectra of ^14^NH_3_ and ^15^NH_3_, respectively. ^1^H NMR allows for quick sample preparation, but unless expensive spectrometers are available, the sensitivity is limited which leads to either a long electrolysis time to accumulate enough ammonia to reach the detection limit or a long analysis time per sample to acquire and average enough scans to increase the detection limit sufficiently. Adding Gd^3+^ to the NMR solution as a paramagnetic relaxation agent increases the sensitivity, but even then, 17 μM NH_3_ must be reached for quantitative analysis (400 MHz NMR, no cryoprobe, 15 min analysis time per sample).^29^ In accordance with Figure 1c, reaching 17 μM NH_3_ with a catholyte volume of 30 mL takes 1.4 h. The long electrolysis time in NRR studies is therefore not only caused by NH_3_ contamination but also by the limited sensitivity of ^1^H NMR. We note that the extent to which both of these factors limit the electrolysis time depends strongly on a cell parameter, the electrolyte volume.

Several reasons make NH_3_ contaminations so difficult to avoid that some authors believe that no catalyst has been unambiguously proven to be active for NRR in an aqueous electrolyte.^7,8^ NH_3_ contamination can originate from many, often unexpected sources (see Table S2). These can easily look like genuine NRR because the NH_3_ increase can be time dependent (e.g., NH_3_ that slowly leaches from a Nafion membrane) and potential dependent (e.g., NO_*x*_ that gets reduced electrochemically to NH_3_).^8,30^ Some contamination sources can contaminate a whole batch of experiments (e.g., contaminated catalyst precursor) or only a single experiment (e.g., touched electrolyte with a nitrile glove).^7,16^ The identification and elimination of NH_3_ contamination sources should precede any NRR measurement. As early as 2018, Greenlee et al. reported that there is a high risk of false positives in NRR experiments and that there is a gap between what experimental protocols should be like for unambiguous measurements and what is done in practice. They proposed a protocol for unambiguous measurement of NRR activity which is still valid today.^12^ In the following years, several authors reassessed the reliability of NRR research and found that the gap still exists, although it is slightly smaller since more papers include background measurements and at least qualitative ^15^N_2_ experiments.^7,8,14^ Because the paper by Greenlee et al. was published over three years ago, we think that a lack of knowledge about reliable protocols can no longer explain why the gap still exists. Instead, we think there must be practical barriers that prevent the implementation of reliable protocols.

To examine if there are any practical barriers to implementing reliable detection protocols, we examine the most important step of such protocols: the isotope labeling step. All proposed NRR protocols agree that properly executed isotope labeling experiments that quantitatively agree with ^14^N_2_ data are essential for an unambiguous proof of NRR activity.^7,12,14^ We calculate that one experiment with ^15^N_2_ in an H-cell with typical operating conditions (experiment time: 2h, flow rate: 40 mL/min, see Table S3) would cost about €2400 due to the high cost of ^15^N_2_ (≈ €500/L). At this cost, isotope labeling experiments are obviously prohibitively expensive. Some authors try to circumvent this problem by using drastically reduced flow rates,^31^ operating in fed batch mode,^32^ using a static gas atmosphere^33^ or recirculating the gas^34^ during ^15^N_2_ experiments. While reducing or interrupting gas flow reduces cost, Clark et al. showed that a minimum flow rate of 10 mL/min into an 1.6 mL H-cell is necessary to prevent a sharp decrease of the mass transport of the dissolved gas to the electrode surface (quantified by measuring the boundary layer thickness with ferrocyanide reduction).^26^ The reason for the reduced mass transport is that the gas that is bubbled into the cell is a source of convection which helps transport the dissolved gas to the electrode. As the flow rate is reduced, less convection from the gas bubbles leads to lower mass transport of dissolved gas to the electrode surface and an increase in boundary layer thickness as shown in Figure 1d.^26^ To understand if reduced mass transport can be tolerated for NRR, we estimate the mass transport limiting current of NRR in an H-cell from the limiting current of CO_2_RR to CO in an H-cell (10 mA/cm^2^) by taking into account the different solubility and diffusion coefficient of CO_2_ and N_2_ and the different number of electrons involved in each reaction (see eq (1) in the Supporting Information).^26,35−38^ The resulting mass transport limiting current for NRR is ≈0.6 mA/cm^2^, which means that at the upper end of the range of reported NH_3_ production rates (3–300 μA/cm^2^), NRR is most likely already influenced by mass transport limitations.^25^ This is undesirable because mass transport limitations will reduce the ammonia production and make results difficult to reproduce because the transition from activation controlled to mass transfer controlled kinetics is not well-defined in an H-cell.^22^ A further reduction of the mass transport limiting current due to a reduction of the gas flow rate below 10 mL/min can therefore not be tolerated. The sharply decreasing mass transport below 10 mL/min explains why reports with reduced flow rate are unable to achieve quantitative agreement between ^14^N_2_ and ^15^N_2_ data because mass transport limitations will lower the NH_3_ production rate with ^15^N_2_ if too low flow rates are used.^31,32^ Another issue that increases the cost of isotope-labeling in H-cells is that the electrolyte must be presaturated with ^15^N_2_ prior to electrolysis (typically for 30 min) which adds to the cost.^39^ Nielander et al. showed that the cost per experiment can be reduced by recirculating ^15^N_2_, but the remaining cost is still high (€100 per experiment) because the whole volume of the home-build gas recirculation setup must be flushed with ^15^N_2_.^7,34^ Thus, the necessity for flow rates >10 mL/min in H-cells is a fundamental barrier to reliable data collection in an H-cell. None of the available solutions reduces the cost sufficiently to make quantitative ^15^N_2_ control experiments as accessible as they have to be.

The above analysis has shown that while it is possible to reduce the electrolyte volume in H-cells to decrease the electrolysis time, the N_2_ flow rate cannot be reduced below 10 mL/min, which creates an unavoidable cost barrier toward implementation of reliable protocols for NRR research. Unless this limitation is removed, the uncertainties about the reliability of results in the NRR field are unlikely to go away, or the field is set to become exclusive to those who can afford regular isotope labeling. To avoid this, ^15^N_2_ experiments have to become affordable (e.g., around €10 per experiment) and short (15–20 min). In the following section, we explore if these requirements can be implemented with a gas diffusion electrode (GDE) cell design.

**Figure 2:**
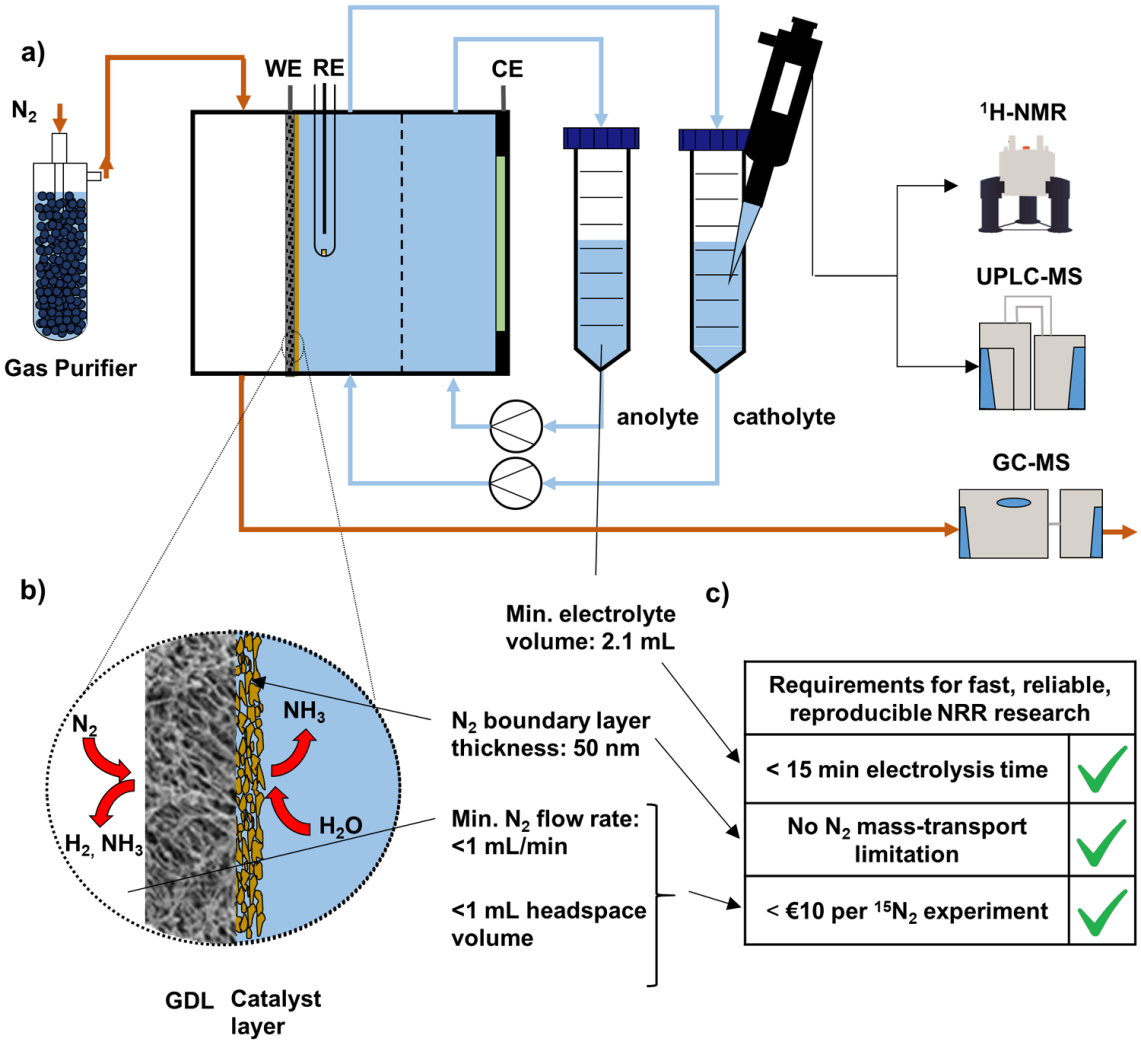
Schematic illustrating how cell parameters of the gas diffusion electrode cell design are influencing reliability and speed of NRR research. (a) Schematic of a gas diffusion electrode cell and NH_3_ detection. (b) Schematic of the surface of a gas diffusion electrode. (c) Checklist for fast, reliable, reproducible NRR research.

A typical gas diffusion electrode cell (see Figure 2a) consists of three compartments. For the NRR, the main difference to H-cells is that in a GDE cell, N_2_ is not bubbled directly into the catholyte but flows past a hydrophobic gas diffusion electrode which separates the catholyte and gas compartment. The catalyst is positioned on the GDE at the interface of the catholyte and gas phase (Figure 2b). The hydrophobicity of the GDE prevents the electrolyte from entering the gas phase. Due to the small distance that the reactant gas has to travel from the gas phase to reach the catalyst (≈50 nm compared to 50 μm in an H-cell) mass transport is much higher than in H-cells.^23^ Therefore, higher mass transport limited current densities for NRR can be reached in GDE cells.^40,41^ Both the anolyte and catholyte are recycled between the cell and a reservoir. During electrolysis insoluble reaction products will enter the gas compartment and leave the cell with the feed gas. Soluble products such as NH_3_ will mostly remain in the electrolyte.^23^

To reach the ^1^H NMR detection threshold of 17 μM NH_3_ in the catholyte within 15 min electrolysis time with an intermediate NH_3_ production rate, the volume of the catholyte should be less than 5 mL (see Figure 1c).^29^ The volume of the catholyte is comprised of 4 parts, the internal volumes of half-cell, reservoir, tubing connections, and peristaltic tubing for the pump. For a standard GDE cell, these volumes can be as low as 0.8, 0.4, 2, and 1.6 mL, respectively, assuming an 8 mm thick catholyte compartment, 20 cm 1/8″ inner diameter (i.d.) peristaltic pump tubing and 1 m 1/16″ i.d. tubing connections.^23^ To reduce this further, the tubing size can be reduced to smaller, commercially available sizes (1/16″ i.d. peristaltic tubing and 1/32″ i.d. tubing for the remaining connections). This leads to volumes of 0.8, 0.4, 0.5, and 0.4 mL, respectively, and a total volume of 2.1 mL which is sufficient to reduce the electrolysis time to less than 15 min.

To reduce the cost of isotope labeling to €10 per experiment, the ^15^N_2_ consumption must be less than 20 mL per experiment. For a 15 min experiment, this means that the flow rate should be less than 1 mL/min (plus 5 mL to flush the system). As discussed above, the flow rate in H-cells must be higher than 10 mL/min for sufficient mass transport of dissolved N_2_ from the bulk electrolyte to the catalyst surface.^26^ On the other hand, in GDE cells, N_2_ has to travel first through a gas-filled gas diffusion layer and then through an electrolyte-filled catalyst layer to reach the surface where the reaction takes place (see Figure 2b). In this configuration, the flow rate of N_2_ can only influence the N_2_ mass transport through the gas phase. However, N_2_ diffuses much faster through gas than through liquid. Therefore, the N_2_ mass transport through the gas phase is not limiting the N_2_ mass transport to the catalyst surface unless very high N_2_ consumption rates are reached. To estimate if the N_2_ flow rate influences N_2_ mass transport at typical N_2_ consumption rates in NRR experiments (<300 μA/cm^2^ NRR partial current density), we draw on experience from the CO_2_RR again.^25^ Tan et al. showed that a GDE cell for CO_2_ reduction (electrode area: 2 cm^2^) can be operated at 200 mA/cm^2^ at flow rates as low as 5 mL/min without observing differences in potential or H_2_ FE compared to higher flow rates.^42^ Since NRR current densities are at least 3 orders of magnitude lower than that, the N_2_ mass transport in GDE cells is independent of the N_2_ flow rate in the relevant current density range for NRR experiments. Therefore, flow rates <1 mL/min are possible in GDE cells. To stay below 20 mL total ^15^N_2_ consumption, the total headspace of the system should be minimal (<1 mL) so that 5 mL ^15^N_2_ are sufficient to flush the system before starting a ^15^N_2_ experiment. The total headspace is comprised of the volumes of the gas compartment of the cell and the headspace of the purifier to remove contaminations from the feed gas and tubing connections. State of the art flow fields have a flow channel thickness of around 1 mm and 20 cm 1/32″ i.d. tubing should be sufficient for the connections which adds only 100 μL to the headspace, respectively.^43^ For proper isotope labeling experiments, it is crucial that NO_*x*_ is effectively removed from the incoming gas stream because especially ^15^N_2_ is likely to be contaminated with NH_3_/NO_*x*_ (see Table S2). NH_3_/NO_*x*_ impurities can be removed with little additional headspace using impurity traps filled with Cu–Zn–Al oxide catalysts as described by Andersen et al. or with a miniaturized version of the oxidizing trap proposed by Choi et al. using an alkaline KMnO_4_ solution (see Figure S1).^7,8^ Both purifier types only add a few 100 μL to the headspace so that the total headspace is sufficiently small for €10 isotope labeling experiments. In summary, all requirements for fast, reliable, reproducible NRR research shown in Figure 2c can be fulfilled with GDE cells.

We want to briefly highlight the opportunity of combining the low isotope labeling cost in a GDE cell with very sensitive ^1^H NMR spectrometers or the recently developed, highly sensitive detection methods for aqueous and gaseous ammonia detection using ultrahigh performance liquid chromatography-mass spectrometry (UPLC-MS) and gas chromatography-mass spectrometry (GC-MS), respectively (see Figure 2a).^24,44,45^ Unlike ^14^NH_3,_^15^NH_3_ is not affected by contaminations, other than the ones coming from the ^15^N_2_ itself which can be removed with a proper gas purification step. Therefore, ^15^NH_3_ from NRR can be quantified accurately as soon as the detection limit of the detection method is reached which is 1 ppm for GC-MS and less than 1 μM for very sensitive ^1^H NMR spectrometers and UPLC-MS, respectively_._^24,44,45^ By using these very sensitive detection methods, ^15^N_2_ experiments can become even shorter and cheaper so that catalysts could potentially be tested only with ^15^N_2_ instead of ^14^N_2_ requiring only a few milliliters of ^15^N_2_ per catalyst and consequentially enabling rapid, unambiguous NH_3_ quantification. With GC-MS, gaseous ^14^NH_3_/^15^NH_3_ can be detected in operando with no external sample manipulations and at very low NH_3_ production rates (on the order of 10^–13^ mol/s at 1 mL/min) which makes the detection more reliable and more sensitive than the commonly used NH_3_ accumulation in an acid trap.^44^

### Electrochemical Benefits of a High Surface Area NRR Catalyst

The choice of cell design has several other implications for NRR research besides the ones discussed in the previous section. An important implication to consider when switching from the H-cell to the GDE cell is that the electrode changes from a low electrochemical active surface area (ECSA) 2D electrode to a high ECSA 3D electrode. In the following, we want to discuss the implications of this transition for NRR research.

**Figure 3:**
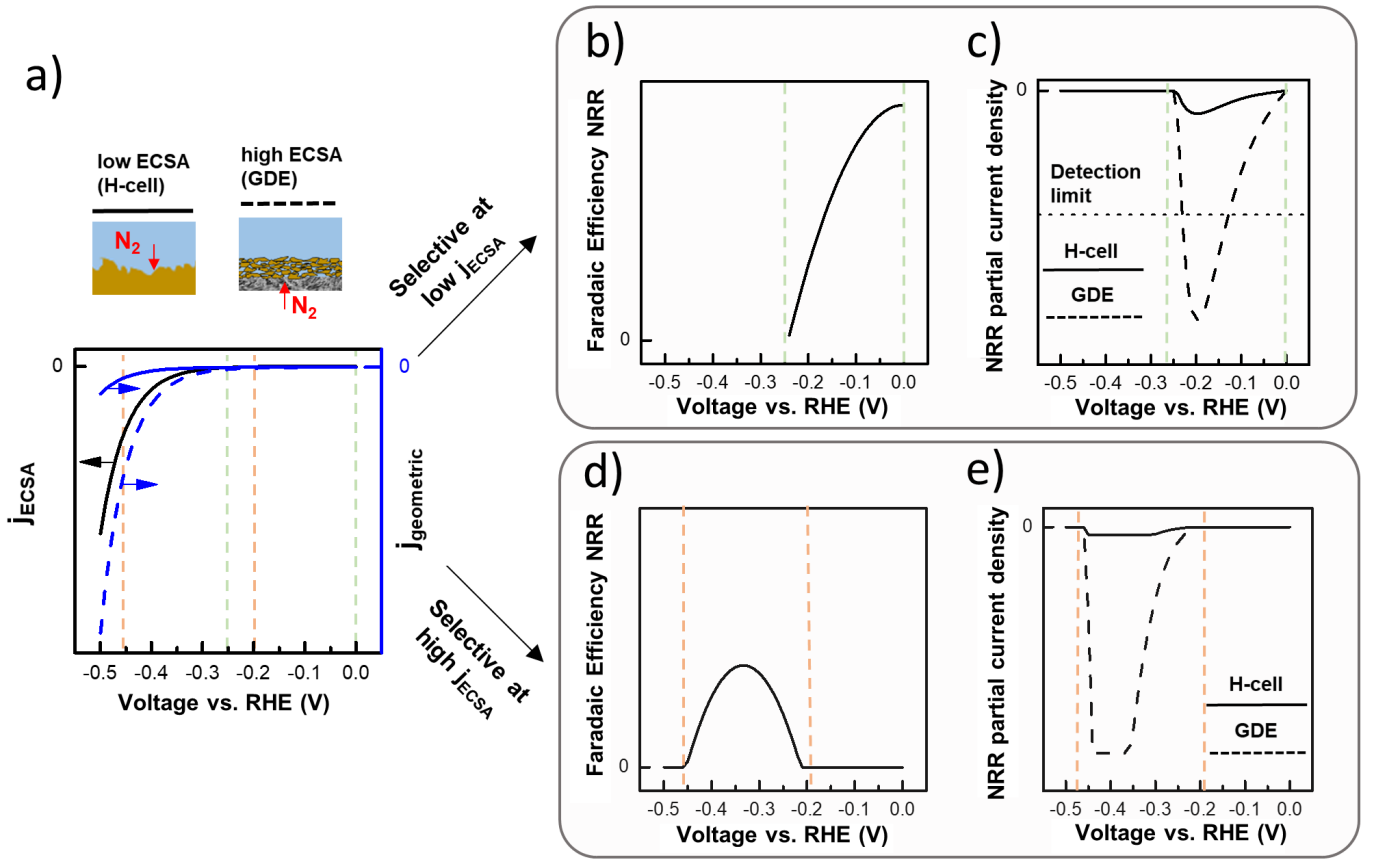
Comparative activity of different surface area systems assuming the same specific activity and faradaic efficiency. (a) Specific activity (ECSA-normalized, black) and resulting current density normalized by geometric surface area (blue) for low-ECSA and high-ECSA electrode. (b,d) Assumed faradaic efficiency if NRR is favorable over HER at low/high specific activity, respectively. (c,e) NRR partial current density assuming the specific activity in (a) and the faradaic efficiency in (b,d), respectively. We assumed that the high-ECSA electrode has a 30-fold higher mass-transport limiting current than the low-ECSA electrode. Specific activity and faradaic efficiency were modeled by using the Butler–Volmer equation and quadratic functions, respectively.

Three dimensional (3D) nanostructured electrodes such as GDEs have a 10- to 1000-fold higher roughness factor (defined as ECSA available for nitrogen reduction normalized by geometric surface area) than two-dimensional (2D) electrodes which are commonly used in H-cells.^5,22^ It is noteworthy that even if an electrode with a 3D morphology is used in an H-cell, the active surface area for NRR will be approximately 2D because the active sites in the bulk of the electrode are insufficiently supplied with nitrogen at very low current densities. Not only is most of the catalyst layer then not active for NRR but there are much larger ECSA’s available for HER than NRR.^23^ To understand how the roughness factor of an electrode can influence selectivity and product detection, we compare the NH_3_ production of two electrode configurations, one 2D electrode in an H-cell and one 3D electrode in a GDE cell. We assume a 10-fold higher roughness factor for the 3D electrode in the GDE cell and that both electrode configurations have the same specific activity (i.e., ECSA normalized current density). As shown in Figure 3a, due to its higher surface area, the current density normalized by the geometric surface area is higher for the 3D GDE than for the 2D H-cell electrode. We include ammonia production into the model by assuming that the kinetically possible NRR faradaic efficiency (i.e., no mass transport limitation) can be described with a parabolic function. We distinguish two cases in the model of the faradaic efficiency. In the first case, we assume that a potential window where NRR is selective exists at low specific activity (Figure 3b). In the second case, we assume that the selective potential window exists at high specific activity (Figure 3d).

For the first case, we show in Figure 3c what happens to the ammonia production when using a low/high ECSA electrode, respectively. As we showed in the previous section, there is a minimum amount of NH_3_ which has to be produced by the catalyst for the NRR activity to be detectable and distinguishable from contamination. In Figure 3c, we assume that the ammonia production of the 2D electrode in an H-cell is too low to reach this detection limit and hence the NRR activity will not be discoverable. On the other hand, with a 3D GDE, larger current densities (and therefore larger ammonia production rates) can be reached at lower overpotentials due to its higher ECSA. In consequence, the NRR activity which was previously undiscoverable in an H-cell becomes discoverable in a GDE cell. Therefore, using 3D GDE’s instead of 2D electrodes in an H-cell makes it possible to measure the selectivity of materials at lower overpotentials. Testing materials in this low overpotential region might yield catalysts with improved selectivity. For example, in the CO_2_RR scientific field, a shift toward more desirable product distributions was discovered by testing materials in a GDE cell which is believed to be caused by the fact that higher current densities can be reached at lower overpotentials.^5^

Figure 3e shows the ammonia production in the case that a selective potential window exists at high specific activity. In this case, the corresponding NH_3_ production is high enough for mass transport limitations to play a role. We assume a mass transport limiting current that is 30-fold higher for the 3D electrode than for the 2D electrode corresponding to the approximate difference in mass transport between the H-cell and the GDE cell.^26,46^ Both electrodes do not reach their kinetically possible maximum faradaic efficiency due to mass transport limitations. However, the faradaic efficiency is higher for the 3D GDE because the mass transport limitation occurs at higher currents. Therefore, using 3D GDEs can increase the selectivity compared to 2D electrodes in an H-cell in cases where the latter is operated in the mass transport limited current region. The potential of GDE’s to achieve high NH_3_ production rates by circumventing N_2_ mass transport limitations has been demonstrated by Lazouski et al. who showed that NH_3_ partial current densities up to 8.8 mA/cm^2^ can be obtained using a lithium-mediated approach with stainless steel gas diffusion electrodes.^47^

Stable catalysts are essential when using detection methods that rely on the accumulation of NH_3_ because if a catalyst deactivates before the threshold NH_3_ concentration is reached, it will not be detectable. The stability of a catalyst can be compromised by impurity deposition onto its surface, surface reconstruction, and morphology changes.^22^ In NRR experiments, the risk of impurity deposition on the electrode surface is particularly high because over long electrolysis times, high negative overpotentials and alkaline electrolytes are used which increase the risk of impurity deposition.^39^ This risk can be reduced by using high ECSA GDE’s because their higher ECSA reduces the fraction of the surface that can be affected by impurity deposition for a fixed amount of impurities. Furthermore, impurities will deposit preferably on the side of the electrode that is facing the electrolyte, not on the N_2_ side of the GDE where NRR can be expected to take place preferably.^21,22,39^ Surface reconstruction and morphology changes might also affect high ECSA electrodes less, because the overpotential to reach a certain current density will be lower which might reduce the magnitude of such effects.^46^

### Parallel Examples of GDEs as a Benchmarking Cell Design

A benchmark consists of a clearly defined electrochemical setup and a set of protocols describing how to carry out a measurement with a well-defined catalyst to reproduce a known catalyst performance. Benchmarks are useful when developing electrocatalysts because they ensure the reliability and reproducibility that is necessary to evaluate and compare new catalysts unambiguously.^48^ Currently, the NRR academic community has no benchmarking materials or protocols because there is no generally accepted catalyst for this reaction yet. However, eventually a benchmark will have to be developed for NRR because it can expedite catalyst development. To understand what a suitable benchmark for NRR might look like we will briefly look at how benchmarks are performed in comparable electrochemical fields with low solubility gaseous reagents.

In the case of the oxygen reduction reaction (ORR), specific values for mass-transport limiting current and mass activity must be reached with a Pt/C catalyst in a rotating disk electrode (RDE) setup to confirm that the setup is comparable to literature.^39,48^ However, as recent results have shown, it is not always possible to transfer the activity of promising catalysts measured at low current density in RDE setups to high current density commercial devices.^49^ For example, nanostructured Pt-based ORR catalysts such as Pt–Ni nanoframes have much lower mass activity under real fuel cell conditions than predicted by low current density RDE measurements.^49^ Similarly, for a long time, CO_2_RR catalysts were compared at low current density in H-cells, but when those catalysts were tested at higher current density in GDE cells, they had completely different product distributions.^46,50^ The lack of transferability of results can arise from a variety of changes that occur when catalysts are tested in commercial devices instead of low current density catalyst testing devices, for example, changes in local mass transport, pH, or catalyst layer quality.^23,51^ A possible solution to this problem would be to benchmark catalysts in membrane electrode assemblies (MEA) where they can be tested at high current density. However, the production of MEAs is time-consuming, and it is challenging to control temperature, pressure, water distribution, and prevent gas crossover.^52,53^ Therefore, in both fields, CO_2_RR and ORR, GDE cells have emerged as an alternative platform to test catalysts at current densities closer to commercial conditions but without the problems associated with using a MEA cell design. For example, Inaba et al. has shown that similar ORR mass activities can be observed in GDE and MEA cell design for a given Pt/C catalyst.^46,54^ Leapfrogging low current density catalyst development and directly adopting a GDE cell design for a NRR catalyst search might prevent years and resources spent recording data at low current density which might not be transferrable to commercial devices.

However, the use of GDE cells to benchmark catalysts instead of H-cells or RDE cells has several disadvantages. Using a GDE cell instead of an H-cell can cause practical problems, for example with the electrical contact or the sealing of the GDE. A description of how to deal with such problems goes beyond the scope of this perspective but interested readers are referred to the relevant literature.^55^ Additionally, a GDE is an ill-defined 3D nanostructure which can have an inhomogeneous distribution of pH and N_2_ concentration due to highly overlapping diffusion gradients. Inside the 3D structure of a GDE many different morphological factors such as grain, porosity, oxidation state, etc. might be superimposed which makes it difficult to extract structure–functionality relationships between morphological factors and intrinsic activity. Due to these implications, GDE’s might not be suitable for fundamental studies where the goal is to measure intrinsic values for activity and selectivity. For such studies H-cells with a well-defined catalyst surface might be a better platform.^5,22^

## Conclusions

The poor reliability and experimental throughput of NRR research is linked to the H-cell-type cell design, with its commonly high electrolyte volume and N_2_ flow rates. These limitations can be overcome by using GDE cells, because mass transport and gaseous flow rate are decoupled resulting in short (<15 min) and cheap (less than €10 per experiment) isotope labeling experiments. The higher ECSA of 3D nanostructured GDEs enables higher NH_3_ production at lower overpotentials and reduces the risk of catalyst deactivation. However, it is less suitable for fundamental or mechanistic studies aiming to measure intrinsic activity/selectivity values because the surface of the catalyst is ill-defined. Leapfrogging to GDE cell design for NRR catalyst development will reduce the uncertainty associated with transferring low current density H-cell data to high current density commercial devices. Because the primary objective of NRR research at the moment is to reliably identify a selective catalyst, the advantages of catalyst development in a GDE cell design clearly outweigh its limitations.

## Supporting Information Available

Description of miniaturized alkaline impurity trap, calculations, and summaries of reported levels of NH_3_/NO_*x*_ contamination and experimental parameters used in NRR literature (PDF)
