# Supplementary material for: Overcoming Nitrogen Reduction to Ammonia Detection Challenges: The Case for Leapfrogging to Gas Diffusion Electrode Platforms
Source: ACS Catal. 2022 Apr 28;12(10):5726–35. doi: 10.1021/acscatal.2c00888 (PMC9127788; doi:10.1021/acscatal.2c00888)
Supplement: Supplementary file 1 — cs2c00888_si_001.pdf [file cs2c00888_si_001.pdf]

Supporting Information: Overcoming nitrogen reduction to ammonia detection challenges: The case for leapfrogging to gas diffusion electrode platforms

Martin Kolen[a]*, Davide Ripepi[a], Wilson A. Smith[a], Thomas Burdyny[a], Fokko M. Mulder,[a]*

[a] Materials for Energy Conversion and Storage (MECS), Department of Chemical Engineering, Faculty of Applied Sciences, Delft University of Technology, van der Maasweg 9, 2629 HZ Delft, The Netherlands

Corresponding Author: m.kolen@tudelft.nl; f.m.mulder@tudelft.nl

Miniaturized alkaline impurity trap

The removal of NH_3 and NO_x contamination from the gas feed of the cell is crucial to avoid false positives while testing catalysts for NRR. Purifiers to achieve this typically consist of an inner and an outer tube. The inner tube is immersed into the outer tube which is filled with an oxidizing solution that traps the NH_3/NO_x. During operation, gas is bubbled into the oxidizing solution through the inner tube. The gas exits the outer tube through its headspace. Typically, both outer tube and inner tube are made of glass, because glass is very inert and easy to reshape. However, the smallest commercially available impurity traps of this design have several mL of headspace volume which would make them very expensive to flush during a $^{15}N_2$ experiment. Therefore, we propose to use an impurity trap made from inert polymers instead, as shown in Figure S1. The working principle of the design is identical to that of glass impurity traps but the inner and outer tube are made of inert polymer tubing. The headspace of the 1/32" outer diameter (OD) inner tubing and the tee is negligible so that the total headspace of the purifier can be estimated from the headspace of the ¼" outer diameter (OD) outer tube. In our experience approximately 1 cm of headspace in the outer tube is sufficient to prevent liquid from entering the gas channel. With an inner diameter (ID) of 5.6 mm the headspace of the outer tube is approximately 250 µL. This low headspace makes it ideal for cheap $^{15}N_2$ experiments in GDE cell. Additionally, it is comprised of standard connectors for easy, leak-tight, contamination-free connections. Unlike with glass impurity traps, it is possible to easily adjust the length of the outer tube depending on the required removal efficiency. The trap only consists of readily available, off-the-shelf parts which should improve standardization of this critical component.

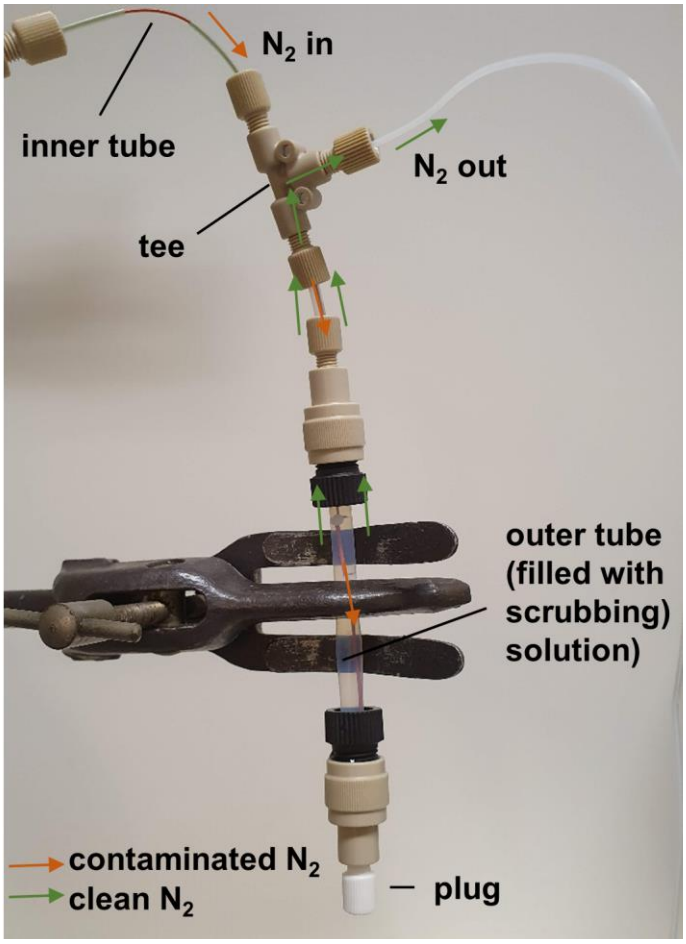

Figure S1 Photograph of a miniaturized purifier to clean NH_3/NO_x contaminated gas streams with minimal additional headspace.

Table S1. Order list for an impurity trap with low headspace volume.

Part #	Name	Quantity	Supplier	Price ($)
1648	Tefzel™ (ETFE) Tubing Natural 1/8" OD x .093" ID x 5ft	1	IDEX Health	33.75
U-665	Adapter Assembly 1/2-20 Female x1/4-28 Female	2	IDEX Health	37.50
P-713	PEEK Low PressureTee Assembly 1/8" PEEK .050 thru hole	1	IDEX Health	38.63
F-247	NanoTight™ Sleeve Green 1/16" OD x .033" ID x 1.6"	4	IDEX Health	2.84
1569	PEEK Tubing Orange 1/32" OD x .020" ID x 5ft	1	IDEX Health	56.93
P-703	Union Assembly PEEK .050 thru hole, for 1/8" OD	2	IDEX Health	24.98
P-249	Super Flangeless™ One-Piece Fitting, 1/4-28 Flat-Bottom, for 1/16" OD	6	IDEX Health	11.02
P-311	Plug Tefzel™ (ETFE) - 1/4-28	1	IDEX Health	2.49
20533	PTFE tubing tubing L × OD × ID 25 ft × 1/4 in. (6.35 mm) × 0.228 in. (5.8 mm)	1	Merck Sigma	88.8

Calculation: nitrogen reduction reaction mass-transport limiting current in H-cell

We estimate the mass transport limiting current of the nitrogen reduction reaction $j_{lim,NRR}$ from the mass-transport limiting current of the CO_2 reduction reaction to CO $j_{lim,CO2RR}$ according to:

$$j_{lim,NRR} \approx j_{\text{lim},CO2RR} \frac{S_{N_2} D_{N_2} z_{NRR}}{S_{CO_2} D_{CO_2} z_{CO2RR}} \tag{1}$$

$$= 10 \frac{mA}{cm^2} \frac{1.27\ x\ 10^{-5}\ x\ 1.77\ x\ 10^{-5} \frac{cm^2}{s} x\ 6}{7\ x\ 10^{-4}\ x\ 1.67\ x\ 10^{-5} \frac{cm^2}{s} x\ 2}$$

$$= 0.6 \frac{mA}{cm^2}$$

, where S_i, D_i, are the solubility and diffusion coefficient of nitrogen and carbon dioxide in water, respectively and z_{NRR}, z_{CO2RR} are the number of electrons transferred in the electrochemical reduction of nitrogen to ammonia and of carbon dioxide to carbon monoxide per molecule of N_2/CO_2, respectively.[1–5]

Calculation: accumulated NH3 in the electrolyte

The concentration of NH_3 in the electrolyte was calculated according to:

$$c_{NH_3} = \frac{i_{NH_3} t}{zFV} \tag{2}$$

, where c_{NH3} is the concentration of NH_3 after electrolysis, i_{NH3} is the partial current density of NRR, *t* is the duration of the experiment, *z* is the number of electrons transferred per molecule of NH_3 produced, F is the Faraday Constant and V is the half-cell volume of the electrolyte, respectively.

Mathematical modelling of influence of ECSA on ammonia production and faradaic efficiency

The specific activity (defined as the ECSA normalized current density) j_{ECSA} was calculated by assuming Butler-Volmer kinetics according to:

$$j_{ECSA} = j_0(e^{-\alpha f \eta} - e^{(1-\alpha) f \eta}) \quad (3)$$

, where j_0 is the exchange current density, α is the symmetry factor, f is the Faraday Constant F divided by the ideal gas constant R and the temperature T and η is the overpotential.[6] The current density normalized by geometric surface area $j_{geometric}$ was calculated by multiplying j_{ECSA} with the roughness factor of the electrode.

The faradaic efficiency was modelled by assuming that a potential window exists where NRR is favorable over HER and that the faradaic efficiency (FE) of NRR within this potential window can be described by a quadratic function:

$$FE = a\eta^2 + b\eta + c \quad (4)$$

, where a,b,c are constant parameters.

The partial current density of NRR j_{NRR} was calculated by multiplying the faradaic efficiency with $j_{geometric}$. The partial current density of NRR including mass transport effects $j_{mt,NRR}$ was calculated by replacing j_{NRR} with the mass transport limiting current $j_{lim,NRR}$ wherever j_{NRR} would otherwise have been lower than $j_{lim,NRR}$:

$$j_{mt,NRR} = \max(j_{NRR}, j_{lim,NRR}) \quad (5)$$

Literature summary of reported levels of NH_3/NO_x contamination

*Table S2. Literature summary of ammonia and nitrate contaminations observed in nitrogen reduction studies. *To make reports more comparable, contaminations reported as absolute amounts in nmol or as concentrations in a gas stream were converted into concentrations in the electrolyte by assuming the following parameters: electrolyte volume 10 mL, catalyst area: 1 cm^2, catalyst amount: 1 mg, gas flow rate: 30 mL/min duration of gas flow: 2h.*

Contamination source	NH_3/NO_x concentration	Reference
Human Breath	0.3-3 ppm NH_3 in gas	[7]
Human Breath	0.28-1.4 ppm NH_3 in gas	[8]
0.05 M H_2SO_4 open to air for 1h	1.7 µM NH_3	[9]
0.05 M H_2SO_4 sealed for 1h	0.6 µM NH_3	[9]
DI water open to air for 400 min	8.8 µM NH_3	[10]
Nitrile gloves sonicated for 1h in DI water	155.1 µM NH_3	[7]
Polypropylene sample storage container (initially)	0.3 - 49.8 µM NO_x	[11]
Polypropylene sample storage container (after 10 days)	3.4 - 65.3 µM NO_x	[11]
Cell, electrolyte, epoxy, electrodes	18.9-21.5 µM NO_x	[11]
N impurities in CoMo film	10 µM NH_3	[11]

Rubber septa	630 µM NH_3, 270 µM NO_x	[12]
Ar	1.3 ppb NO_x in gas (0.02 µM NO_x)*	[11]
N_2	3.1 ppb NO_x in gas(0.046 µM NO_x)*	[11]
Ar, N_2, $^{15}N_2$	200ppb N_2O in gas (3 µM NO_x)*	[13]
$^{15}N_2$	0.024 - 420 ppm $^{15}NO_x$ in gas (0.36-6290 µM $^{15}NO_x$)*<br>0.014 - 1900 ppm $^{15}NH_3$ in gas (0.21 – 28454 µM $^{15}NH_3$)*	[14]
Bi_2O_3, Al_2O_3, Fe_2O_3	10-120 µmol/g catalyst NO_x (1-12 µM NO_x)*	[15]
Commercial metallic iron	16343 – 406469 µM total N	[15]
$^{15}N_2$ scrubbing solution	18.5 µM $^{14}NH_3$, 33 µM $^{15}NH_3$	[11]
Potential induced generation of NH_3 from Fe loaded onto stainless steel	10 µM NH_3	[13]
Release of NH_3 from Nafion 117 membrane soaked in electrolyte containing 0.1 µg/mL of NH_3	17.6 µM NH_3	[16]
Background	1.5 µM NH_3	[17]
Background	2 µM NO_x	[13]
Background	0.5 µM NH_3	[7]

Literature summary of experimental parameters used during aqueous nitrogen electroreduction experiments

Table S3. Literature summary of experimental parameters used during aqueous NRR studies.

Ref	Gas flow rate (mL/min)	Electrolyte volume (mL)	Electrode Area (cm^2)	Electrolysis time (h)	^{15}N? [a]	QT? [b]	Flow rate ^{15}N (ml/min)	Electrolysis time ^{15}N (h)
[18]	n.a.	n.a.	1	2	yes	no	n.a.	10
[19]	0.48	100	n.a.	2	yes	no	0.48	2
[20]	250	n.a.	6.25	1	yes	no	5	1
[21]	n.a.	30	1	n.a.	yes	no	n.a.	2
[22]	n.a.	30	1	2	no	no	n.a.	n.a.
[23]	n.a.	90	2	6	yes	no	20 mL every 10 min	6
[24]	no flow?	30	2	2	yes	no	n.a.	2
[25]	20	n.a.	n.a.	2	yes	no	n.a.	n.a.
[26]	n.a.	50	1	2	yes	no	n.a.	2
[27]	n.a.	n.a.	1	n.a.	no	no	n.a.	n.a.
[28]	n.a.	n.a.	1.5	n.a.	no	no	n.a.	n.a.
[29]	n.a.	30	1	2	yes	yes	n.a.	10
[30]	n.a.	n.a.	1	2	no	no	n.a.	n.a.
[31]	n.a.	35	1	2	no	no	n.a.	n.a.
[32]	n.a.	25	1	n.a.	yes	no	20 mL every 10 min	6
[33]	10	30	0.07	3	yes	no	n.a.	n.a.

34	60	10	n.a.	n.a.	yes	yes	static	0.5
$\tilde{x}$	40	30	1	2				

[a] ^{15}N?: Were control experiments with ^{15}N performed?
[b] QT?: Was a quantitative agreement between $^{14}NH_3$ and $^{15}NH_3$ data demonstrated?

References

(1) Battino, R.; Rettich, T. R.; Tominaga, T. The Solubility of Nitrogen and Air in Liquids. *Journal of Physical and Chemical Reference Data* **1984**, *13* (2), 563–600. https://doi.org/10.1063/1.555713.

(2) Ferrell, R. T.; Himmelblau, D. M. Diffusion Coeffkients of Nitrogen and Oxygen in Water. **1967**, *12* (1), 5. https://doi.org/10.1021/je60032a036.

(3) Carroll, J. J.; Slupsky, J. D.; Mather, A. E. The Solubility of Carbon Dioxide in Water at Low Pressure. *Journal of Physical and Chemical Reference Data* **1991**, *20* (6), 1201–1209. https://doi.org/10.1063/1.555900.

(4) Jähne, B.; Heinz, G.; Dietrich, W. Measurement of the Diffusion Coefficients of Sparingly Soluble Gases in Water. *Journal of Geophysical Research* **1987**, *92* (C10), 10767. https://doi.org/10.1029/JC092iC10p10767.

(5) Clark, E. L.; Resasco, J.; Landers, A.; Lin, J.; Chung, L.-T.; Walton, A.; Hahn, C.; Jaramillo, T. F.; Bell, A. T. Standards and Protocols for Data Acquisition and Reporting for Studies of the Electrochemical Reduction of Carbon Dioxide. *ACS Catalysis* **2018**, *8* (7), 6560–6570. https://doi.org/10.1021/acscatal.8b01340.

(6) Bard, A. J.; Faulkner, L. R. In *Electrochemical methods: fundamentals and applications*; Wiley: New York, 2001; p 96.

(7) Andersen, S. Z.; Čolić, V.; Yang, S.; Schwalbe, J. A.; Nielander, A. C.; McEnaney, J. M.; Enemark-Rasmussen, K.; Baker, J. G.; Singh, A. R.; Rohr, B. A.; Statt, M. J.; Blair, S. J.; Mezzavilla, S.; Kibsgaard, J.; Vesborg, P. C. K.; Cargnello, M.; Bent, S. F.; Jaramillo, T. F.; Stephens, I. E. L.; Nørskov, J. K.; Chorkendorff, I. A Rigorous Electrochemical Ammonia Synthesis Protocol with Quantitative Isotope Measurements. *Nature* **2019**, *570* (7762), 504–508. https://doi.org/10.1038/s41586-019-1260-x.

(8) Larson, T. V.; Covert, D. S.; Frank, R.; Charlson, R. J. Ammonia in the Human Airways: Neutralization of Inspired Acid Sulfate Aerosols. *Science* **1977**, *197* (4299), 161–163. https://doi.org/10.1126/science.877545.

(9) Duan, G. Y.; Ren, Y.; Tang, Y.; Sun, Y. Z.; Chen, Y. M.; Wan, P. Y.; Yang, X. J. Improving the Reliability and Accuracy of Ammonia Quantification in Electro- and Photochemical Synthesis. *ChemSusChem* **2020**, *13* (1), 88–96. https://doi.org/10.1002/cssc.201901623.

(10) Saigne, C.; Kirchner, S.; Legrand, M. ION-CHROMATOGRAPHIC MEASUREMENTS OF AMMONIUM, FLUORIDE, ACETATE, FORMATE AND METHANESULPHONATE IONS AT VERY LOW LEVELS IN ANTARCTIC ICE. *Analytica Chimica Acta,* **1987**, *203*, 11–21.

(11) Yu, W.; Buabthong, P.; Read, C. G.; Dalleska, N. F.; Lewis, N. S.; Lewerenz, H.-J.; Gray, H. B.; Brinkert, K. Cathodic NH_4^+ Leaching of Nitrogen Impurities in CoMo Thin-Film Electrodes in Aqueous Acidic Solutions. *Sustainable Energy & Fuels* **2020**, *4* (10), 5080–5087. https://doi.org/10.1039/D0SE00674B.

(12) Boucher, D. L.; Davies, J. A.; Edwards, J. G.; Mennad, A. An Investigation of the Putative Photosynthesis of Ammonia on Iron-Doped Titania and Other Metal Oxides. *Journal of Photochemistry and Photobiology A: Chemistry* **1995**, *88* (1), 53–64. https://doi.org/10.1016/1010-6030(94)03994-6.

(13) Hodgetts, R.; Du, H.-L.; MacFarlane, D. R.; Simonov, A. N. Electrochemically Induced Generation of Extraneous Nitrite and Ammonia in Organic Electrolyte Solutions during Nitrogen Reduction

Experiments. *ChemElectroChem* **2021**, *8* (9), 1596–1604. https://doi.org/10.1002/celc.202100251.
(14) Dabundo, R.; Lehmann, M. F.; Treibergs, L.; Tobias, C. R.; Altabet, M. A.; Moisander, P. H.; Granger, J. The Contamination of Commercial 15N2 Gas Stocks with 15N–Labeled Nitrate and Ammonium and Consequences for Nitrogen Fixation Measurements. *PLoS ONE* **2014**, *9* (10), e110335. https://doi.org/10.1371/journal.pone.0110335.
(15) Chen, Y.; Liu, H.; Ha, N.; Licht, S.; Gu, S.; Li, W. Revealing Nitrogen-Containing Species in Commercial Catalysts Used for Ammonia Electrosynthesis. *Nat Catal* **2020**, *3* (12), 1055–1061. https://doi.org/10.1038/s41929-020-00527-4.
(16) Ren, Y.; Yu, C.; Tan, X.; Han, X.; Huang, H.; Huang, H.; Qiu, J. Is It Appropriate to Use the Nafion Membrane in Electrocatalytic N_2 Reduction? *Small Methods* **2019**, *3* (12), 1900474. https://doi.org/10.1002/smtd.201900474.
(17) Yu, W.; Lewis, N. S.; Gray, H. B.; Dalleska, N. F. Isotopically Selective Quantification by UPLC-MS of Aqueous Ammonia at Submicromolar Concentrations Using Dansyl Chloride Derivatization. *ACS Energy Letters* **2020**, *5* (5), 1532–1536. https://doi.org/10.1021/acsenergylett.0c00496.
(18) Zhao, L.; Liu, X.; Zhang, S.; Zhao, J.; Xu, X.; Du, Y.; Sun, X.; Zhang, N.; Zhang, Y.; Ren, X.; Wei, Q. Rational Design of Bimetallic $Rh_{0.6}Ru_{0.4}$ Nanoalloys for Enhanced Nitrogen Reduction Electrocatalysis under Mild Conditions. *Journal of Materials Chemistry A* **2021**, *9* (1), 259–263. https://doi.org/10.1039/D0TA09099A.
(19) Wei, X.; Vogel, D.; Keller, L.; Kriescher, S.; Wessling, M. Microtubular Gas Diffusion Electrode Based on Ruthenium-Carbon Nanotubes for Ambient Electrochemical Nitrogen Reduction to Ammonia. *ChemElectroChem* **2020**, *7* (22), 4679–4684. https://doi.org/10.1002/celc.202001370.
(20) Kim, M.-C.; Nam, H.; Choi, J.; Kim, H. S.; Lee, H. W.; Kim, D.; Kong, J.; Han, S. S.; Lee, S. Y.; Park, H. S. Hydrogen Bonding-Mediated Enhancement of Bioinspired Electrochemical Nitrogen Reduction on $Cu_{2-x}S$ Catalysts. *ACS Catalysis* **2020**, *10* (18), 10577–10584. https://doi.org/10.1021/acscatal.0c01730.
(21) Jin, Y.; Ding, X.; Zhang, L.; Cong, M.; Xu, F.; Wei, Y.; Hao, S.; Gao, Y. Boosting Electrocatalytic Reduction of Nitrogen to Ammonia under Ambient Conditions by Alloy Engineering. *Chemical Communications* **2020**, *56* (77), 11477–11480. https://doi.org/10.1039/D0CC02489A.
(22) Jiang, X.; He, M.; Tang, M.; Zheng, Q.; Xu, C.; Lin, D. Nanostructured Bimetallic Ni–Fe Phosphide Nanoplates as an Electrocatalyst for Efficient N2 Fixation under Ambient Conditions. *J Mater Sci* **2020**, *55* (31), 15252–15262. https://doi.org/10.1007/s10853-020-05085-5.
(23) Lin, Y.-X.; Zhang, S.-N.; Xue, Z.-H.; Zhang, J.-J.; Su, H.; Zhao, T.-J.; Zhai, G.-Y.; Li, X.-H.; Antonietti, M.; Chen, J.-S. Boosting Selective Nitrogen Reduction to Ammonia on Electron-Deficient Copper Nanoparticles. *Nat Commun* **2019**, *10* (1), 4380. https://doi.org/10.1038/s41467-019-12312-4.
(24) Zhang, L.; Cong, M.; Ding, X.; Jin, Y.; Xu, F.; Wang, Y.; Chen, L.; Zhang, L. A Janus Fe-SnO_2 Catalyst That Enables Bifunctional Electrochemical Nitrogen Fixation. *Angew. Chem. Int. Ed.* **2020**, *59* (27), 10888–10893. https://doi.org/10.1002/anie.202003518.
(25) Li, Y.; Chen, J.; Cai, P.; Wen, Z. An Electrochemically Neutralized Energy-Assisted Low-Cost Acid-Alkaline Electrolyzer for Energy-Saving Electrolysis Hydrogen Generation. *Journal of Materials Chemistry A* **2018**, *6* (12), 4948–4954. https://doi.org/10.1039/C7TA10374C.
(26) Zhao, S.; Liu, H.; Qiu, Y.; Liu, S.; Diao, J.; Chang, C.; Si, R.; Guo, X. An Oxygen Vacancy-Rich Two-Dimensional Au/TiO2 Hybrid for Synergistically Enhanced Electrochemical N2 Activation and Reduction. *J. Mater. Chem. A* **2020**, *8* (14), 6586–6596. https://doi.org/10.1039/D0TA00658K.
(27) Wang, J.; Ren, Y.; Chen, M.; Cao, G.; Chen, Z.; Wang, P. Bismuth Hollow Nanospheres for Efficient Electrosynthesis of Ammonia under Ambient Conditions. *Journal of Alloys and Compounds* **2020**, *830*, 154668. https://doi.org/10.1016/j.jallcom.2020.154668.
(28) Tong, Y.; Guo, H.; Liu, D.; Yan, X.; Su, P.; Liang, J.; Zhou, S.; Liu, J.; Lu, G. Q. (Max); Dou, S. X. Vacancy Engineering of Iron-Doped $W_{18}O_{49}$ Nanoreactors for Low-Barrier Electrochemical

Nitrogen Reduction. *Angew. Chem. Int. Ed.* **2020**, *59* (19), 7356–7361. https://doi.org/10.1002/anie.202002029.

(29) Wang, J.; Huang, B.; Ji, Y.; Sun, M.; Wu, T.; Yin, R.; Zhu, X.; Li, Y.; Shao, Q.; Huang, X. A General Strategy to Glassy M-Te (M = Ru, Rh, Ir) Porous Nanorods for Efficient Electrochemical N_2 Fixation. *Adv. Mater.* **2020**, *32* (11), 1907112. https://doi.org/10.1002/adma.201907112.

(30) Wang, F.; Lv, X.; Zhu, X.; Du, J.; Lu, S.; Alshehri, A. A.; Alzahrani, K. A.; Zheng, B.; Sun, X. Bi Nanodendrites for Efficient Electrocatalytic N_2 Fixation to NH_3 under Ambient Conditions. *Chem. Commun.* **2020**, *56* (14), 2107–2110. https://doi.org/10.1039/C9CC09803H.

(31) Ohrelius, M.; Guo, H.; Xian, H.; Yu, G.; Alshehri, A. A.; Alzahrani, K. A.; Li, T.; Andersson, M. Electrochemical Synthesis of Ammonia Based on a Perovskite $LaCrO_3$ Catalyst. *ChemCatChem* **2020**, *12* (3), 731–735. https://doi.org/10.1002/cctc.201901818.

(32) Xue, Z.-H.; Zhang, S.-N.; Lin, Y.-X.; Su, H.; Zhai, G.-Y.; Han, J.-T.; Yu, Q.-Y.; Li, X.-H.; Antonietti, M.; Chen, J.-S. Electrochemical Reduction of N_2 into NH_3 by Donor–Acceptor Couples of Ni and Au Nanoparticles with a 67.8% Faradaic Efficiency. *J. Am. Chem. Soc.* **2019**, *141* (38), 14976–14980. https://doi.org/10.1021/jacs.9b07963.

(33) Song, P.; Wang, H.; Kang, L.; Ran, B.; Song, H.; Wang, R. Electrochemical Nitrogen Reduction to Ammonia at Ambient Conditions on Nitrogen and Phosphorus Co-Doped Porous Carbon. *Chem. Commun.* **2019**, *55* (5), 687–690. https://doi.org/10.1039/C8CC09256G.

(34) Suryanto, B. H. R.; Wang, D.; Azofra, L. M.; Harb, M.; Cavallo, L.; Mitchell, D. R. G.; Chatti, M.; MacFarlane, D. R. MoS2 Polymorphic Engineering Enhances Selectivity in the Electrochemical Reduction of Nitrogen to Ammonia. *ACS Energy Letters* **2019**, *4* (2), 430–435. https://doi.org/10.1021/acsenergylett.8b02257.
